# Knowledge, attitudes, and readiness about critical antimicrobial resistant organisms among healthcare workers at colonial war memorial hospital in Fiji: a pre and post intervention study

**DOI:** 10.1186/s13756-024-01439-9

**Published:** 2024-09-04

**Authors:** Kirsty Buising, Ravi Naidu, Shammi Prasad, Matthew Richards, Savneel Shivam Kumar, Alvina Lata, Ashlyn Datt, Sisilia Assisi Genaro, Timaima Ratusela, Ilisapeci Nabose, Donna Cameron, Ana Suka, Tracey Young-Sharma, Benjamin P Howden, Aneley Getahun Strobel

**Affiliations:** 1 The Royal Melbourne Hospital, 300 Grattan Street, Melbourne, VIC 3052 Australia; 2 Department of Infectious Diseases, The University of Melbourne, The Peter Doherty Institute for Infection and Immunity, 792 Elizabeth Street, Melbourne, VIC 3000 Australia; 3 Ministry of Health and Medical Services, Colonial War Memorial Hospital, Waimanu road, Suva, Fiji; 4 Australian Volunteer International, Pacific People Office, Level 2, BSP Central Street, Suva, Fiji; 5 Microbiological Diagnostic Unit Public Health Laboratory, The University of Melbourne, The Peter Doherty Institute for Infection and Immunity, 792 Elizabeth Street, Melbourne, VIC 3000 Australia

**Keywords:** Antimicrobial resistance, Attitudes, Fiji, Healthcare Workers, Infection control, Knowledge

## Abstract

**Background:**

Gram-negative bacteria resistant to carbapenems are also known as critical antimicrobial resistant organisms. Their emergence at Colonial War Memorial Hospital (CWMH), the largest hospital in Fiji, is a major clinical concern. This study was conducted to determine the knowledge, attitudes, and readiness of healthcare workers (HCW) at CWMH regarding management of patients with infections caused by critical antimicrobial resistant organisms.

**Methods:**

A questionnaire was designed using a Likert scale to assess knowledge, attitudes, and readiness. Two cross-sectional studies were conducted, before and after the implementation of targeted educational activities which were informed by the pre-intervention study findings.

**Results:**

A total of 393 and 420 HCW participated in the pre- and post-intervention studies, respectively. The majority of respondents were female (77.3%) and 18–34 years of age (67%). HCW professional roles included nurses (56.3%), doctors (31.6%), and laboratory personnel (12.2%). In the post-intervention study, significantly more HCW reported having received infection prevention and control (IPC) and antimicrobial resistance education and training (26.8% in pre to 45.5% in post intervention, *p* < 0.001). The majority of nurses and doctors (> 85% to ≥ 95%) were aware of how AMR organisms spread in healthcare settings and knew the IPC measures to prevent transmission of AMR infections including hand hygiene, standard and transmission-based precautions. Attitudes towards AMR were positive, with 84.2% pre intervention and 84.8% of HCW post intervention expressing their willingness to change their work environment to assist with AMR prevention. Perceived readiness to address the problem showed mixed results. Improvements in laboratory AMR surveillance data availability were noted (29.4–52.4%, *p* < 0001). Modest improvement in the hospital’s capacity for outbreak response (44–51.9%, *p* = 0.01), and treatment of AMR infections (38.9–44.4%, *p* = 0.01) was reported.

**Conclusions:**

Our data revealed high levels of staff awareness and knowledge about AMR and IPC. However, readiness for outbreak response and treatment of critical AMR infections requires more attention. Improving AMR prevention and containment in CWMH will likely require sustained and multisectoral interventions with strong administrative commitment.

**Supplementary Information:**

The online version contains supplementary material available at 10.1186/s13756-024-01439-9.

## Background

The emergence and spread of antimicrobial resistance (AMR) is a major public health threat globally [1]. In 2017, the World Health Organization (WHO) published the priority list of multidrug-resistant organisms (MDRO) for research and urgent development of new antimicrobials [2]. The critical AMR priority list includes *Acinetobacter*, *Pseudomonas* and various *Enterobacterales* resistant to carbapenems and other last-line antimicrobials [3, 4]. Infections with critical AMR organisms in resource-limited settings are a major concern as there is inadequate or limited human, financial and infrastructure capacity to detect infections, limited access and high cost of antimicrobials, and inadequate capacity to implement measures to prevent and control them in healthcare settings [5, 6]. While AMR can occur naturally, some of the main factors that accelerate the emergence of AMR in healthcare facilities are misuse or overuse of antimicrobials and poor infection prevention and control (IPC) practices [7, 8]. A lack of knowledge about AMR can be a contributing factor to inappropriate use or overuse of antimicrobials [9]. Multimodal IPC measures are effective to prevent the transmission of critical AMR infections in healthcare settings [10]. Knowledge of healthcare workers (HCW) about these measures and their consistent application during patient care is of paramount importance. Knowledge, attitudes, and practice studies in low- and middle-income countries have previously showed major gaps in knowledge and practices related to AMR and IPC and highlighted the need for more standardized training to raise awareness and improve attitudes and practices [11–14].

Fiji is an island nation in the South Pacific with a population of 884,887 in 2017 [15]. Recent studies from Fiji’s main hospital, Colonial War Memorial Hospital (CWMH) revealed the substantial burden of organisms with critical AMR including carbapenem-resistant *Acinetobacter baumannii* and Enterobacterales [16, 17]. In addition, healthcare-associated infections (HAI) and outbreaks caused by MDRO have previously been reported in CWMH [18–21]. Outbreak investigations identified poor IPC practices and shortages of relevant consumables as the main barriers to IPC compliance [19, 20]. Fiji launched new national IPC guidelines in 2022. Despite managing a high burden of AMR and the recurrent outbreaks of HAI with MDRO, the HCW’s knowledge, attitudes, and perceptions of their overall readiness for the diagnosis, prevention, and control of critical AMR organisms has not been investigated. The uptake and usage of the current IPC guidelines is not known.

As the threat of AMR continues to expand in Fiji, more work is required to support the health system to better detect AMR organisms and implement prompt IPC interventions. Preparing Fiji for Organisms with Critical Antimicrobial Resistance is a project supported by the Australian Government’s Medical Research Future Fund. This project was jointly implemented by the Fiji Ministry of Health and Medical Services and University of Melbourne between May 2022 through December 2023. As part of this project, two cross-sectional studies (pre- and post-intervention of a capacity building program) were conducted to determine the knowledge, attitudes, and readiness of HCW in CWMH about critical AMR and IPC and to assess the changes associated with the introduction of project interventions.

## Methods

### Study site

CWMH is the largest referral hospital in Fiji with 500 beds and over 26,500 admissions in 2015 [22].

There are a total of 1,879 staff of which, 900 are nurses, 200 doctors and 91 laboratory personnel in 2021 (Unpublished-CWMH statistics). The hospital has five IPC officers and a functional IPC committee. The IPC team works in collaboration with all departments to implement IPC activities and monitor compliance to recommended practices. There is antimicrobial stewardship (AMS) team comprised of consultant physicians, pharmacists and laboratory scientists which conducts weekly AMS rounds in the ICU, provide advice on antimicrobial use and review prescriptions of restricted antimicrobials. The hospital has a microbiology laboratory. Culture and antimicrobial susceptibility testing are performed using approved protocols.

### Pre- and post-intervention studies

Two cross-sectional studies were conducted. The pre-intervention study was conducted in July and August 2022 before the commencement of project activities. Twelve months later, after implementation of project activities, a post-intervention study was conducted in September and October 2023.

The study questionnaire was developed through review of existing literature [23–26] and questions were appraised by local and international investigators. The questionnaire was piloted with a small group of HCW (*n* = 11) to assess the validity and reliability. The final questionnaire was divided into three parts. The first part included demographic and professional information (age group, gender, year of service, position, departments, and IPC training). Two additional questions were added in the post-intervention study to gather data on participation in the baseline study and participation in any educational activities regarding IPC and critical AMR in the past 12 months. The questions in part two and three included knowledge, attitudes and readiness questions which required participants to select one answer from a five-point Likert scale. There were 16 knowledge and 10 attitude questions which addressed topics such as AMR surveillance, transmission, prevention, laboratory detection, communication, and IPC management. There were five readiness questions which focused on staff perceptions of the local capacity to detect and respond to outbreaks, surveillance and resources available for AMR case management. The study was promoted to doctors, nurses, and laboratory personnel in person and at departmental meetings. Participation was voluntary and written informed consent was obtained from all respondents.

The sample size calculation was based on estimated baseline knowledge rate of 50%, estimated dropout or incomplete questionnaires rate of 15%, and 10% non-response rate, so the required sample size was 390 participants for each of the pre- and post-intervention groups.

### Interventions

The project was implemented in CWMH from May 2022 through December 2023. Between May and July 2023, the team conducted baseline IPC and laboratory capacity assessments as well as the pre-intervention survey. The critical AMR capacity building activities commenced in early August 2022 and focused on two broad streams: Microbiology laboratory, and IPC. The laboratory interventions included review or development of laboratory standard operating procedures (SOP) to detect antimicrobial resistance, provision of additional laboratory consumables for the detection of carbapenem resistant organisms (CRO) and other critical AMR organisms, and ongoing training and education of laboratory personnel. Microbiology laboratory staff developed procedures to streamline communication with medical and IPC teams for regular and timely reporting of CRO and other critical AMR organisms. IPC interventions involved development of SOP for screening of patients at high risk for CRO colonisation and IPC precautions to manage CRO colonised and infected patients, that were endorsed by the hospital IPC committee. All IPC nurses received training on the new SOP and subsequently conducted regular education sessions for HCW. Training and education sessions included structured training (1–4 h) or small group ward level education sessions (≤ 1 h). Education topics included standard precautions, transmission-based precautions (TBP) for prevention of CRO transmission (including contact precautions at bedside, use of personal protective equipment (PPE) and appropriate CRO patient placement), screening of high-risk groups and case notification. Between August 2022 and August 2023, seven structured IPC and AMR training sessions were conducted and attended by 229 HCW at CWMH. In addition, the local IPC team carried out 43 small group education sessions in various wards which were attended by 620 staff (Additional file 1 Table 1). Audit tools were developed to monitor compliance with IPC recommendations. The IPC team conducted regular contact precautions audits to assess compliance and gave feedback to HCW. (Additional file 1 Table 2). Systematic recording of CRO cases commenced and a line list was regularly updated and shared with treating medical and IPC team. The project also supported CRO outbreak detection and investigation. On site and remote mentorship and regular meetings were held with microbiologists and infection control specialists in Melbourne, Australia.

### Data analysis

Data were entered into Microsoft Excel (version 16.79.2) and analysed using R package (version: 4.3.2). Descriptive statistics was used to analyse the socio-demographic, knowledge, attitudes, and readiness data. All categorical variables were presented in proportion and percentages. The Chi-squared test was used to compare the pre- and post-intervention knowledge, attitudes, and readiness. A *p*-value < 0.05 was considered as statistically significant.

### Ethics

The study received ethics approval from Fiji National Human Research Ethics Committee (ID number 11/2022) and Human Ethics Advisory Group of The University of Melbourne (ID number 2022-23602-30481-7).

## Results

A total of 816 HCW completed the study questionnaires with the overall response rate of 96% (395/410) of those offered the questionnaire in the pre-intervention period and 84.7% (421/497) in the post-intervention study. Three respondents were excluded due to incomplete responses and 393 and 420 respondents in the pre- and post-intervention studies were included in the analysis, respectively.

### Sociodemographic and professional characteristics

In both studies, around two third of respondents were aged 18–34 years (67%), with a female majority (77.3%) (Table 1). Overall, 61% of HCW had over 5 years of work experience. The HCW professional roles included nurses (56.3%), doctors (31.6%), and laboratory personnel (12.2%). Respondents represented various departments including internal medicine (18.2%), surgery (17.9%), paediatrics (15.3%), obstetrics and gynecology (14%). Laboratory departments included hematology (23%), microbiology (22%), serology (13%), and biochemistry (12%). 15% (63/420) of respondents participated in both studies. The pre-and post-intervention study respondents were comparable in terms of age, sex, and professional category. However, work experience significantly differed (Table 1). The pre-intervention respondents had more senior staff, 34.6% with > 10 years of work experience compared to 27.4% in post-intervention study, *p* = 0.02. The shift was mainly among nurses where significantly more nurses with ≤ 1 year of service took part in the post-intervention (11. 6%) compared to pre-intervention study (3.3%), *p* = 0.003. While the proportion of nurses with > 10 years of experience declined from 38.2% in the pre-intervention to 29.6% in post-intervention study (Additional file 1 Table 3).

**Table 1:** Demographic and professional information of healthcare workers

Description	Pre-intervention *N* = 393	Post-intervention *N* = 420	Total *N* = 813	*P* value^‡^
	*n* (%)	*n* (%)	*n* (%)	
**Age group (years)**	(***n*** = **393**)	(***n*** = **419**)	(***n*** = **812**)	*0.65*
18–34	262 (66.7)	282(67.3)	544 (67.0)	
35–44	81 (20.6)	92 (22.0)	173 (21.3)	
≥ 45	50 (12.7)	45(10.7)	95 (11.7)	
**Gender**	(***n*** = **391**)	(***n*** = **419**)	(***n*** = **810**)	*0.25*
Female	309 (79.0)	317 (75.7)	626 (77.3)	
Male	82 (21.0)	102 (24.3)	184 (22.7)	
**Years of service**	(***n*** = **393**)	(***n*** = **419**)	(***n*** = **812**)	*0.02*
≤ 12month	35 (8.9)	62 (14.8)	97 (11.9)	
1–4 years	108 (27.5)	112 (26.7)	220 (27.1)	
5–10 years	114 (29.0)	130 (31.0)	244 (30.0)	
> 10 years	136 (34.6)	115 (27.4)	251 (30.9)	
**Profession**	(***n*** = **386**)	(***n*** = **419**)	(***n*** = **805**)	*0.74*
Nurses	220 (57)	233 (55.6)	453 (56.3)	
Doctors	117 (30.3)	137 (32.7)	254 (31.6)	
Lab personnel	49 (12.7)	49 (11.7)	98 (12.2)	
**Department**	(***n*** = **392**)	(***n*** = **414**)	(***n*** = **806**)	*< 0.001*
Medical	55 (14.0)	92 (22.2)	147 (18.2)	
Surgical	58 (14.8)	86 (20.8)	144 (17.9)	
Paediatrics	76 (19.4)	47 (11.4)	123 (15.3)	
Obs/Gyn	65 (16.6)	48 (11.6)	113 (14)	
Laboratory	49 (12.5)	49 (11.8)	98 (12.2)	
ICU/CCU	46 (11.7)	51 (12.3)	97 (12.0)	
Emergency	26 (6.6)	34 (8.2)	60 (7.4)	
Others*	17 (4.3)	7 (1.7)	24 (3.0)	
**IPC training in the past 12 months**	(***n*** = **392**)	(***n*** = **420**)	(***n*** = **812**)	*< 0.001*
Yes	105 (26.8)	191 (45.5)	296 (36.5)	
No	278 (70.9)	219 (52.1)	497 (61.2)	
I don’t know	9 (2.3)	10 (2.4)	19 (2.3)	
**Received education on critical AMR in the past 12 month**	-	(***n*** = **416**)	(***n*** = **416**)	-
Yes	NA	191 (45.9)	191 (45.9)	
No	NA	222 (53.4)	222 (53.4)	
I don’t know	NA	3 (0.7)	3 (0.7)	

‡Comparison of the pre and post intervention findings, *Others include infection control, clinical governance, oncology, and outpatient clinics, ICU = Intensive care unit, CCU = Cardiac care unit, Obs/Gyn = obstetrics and gynaecology, IPC = infection prevention and control, AMR = antimicrobial resistance

The proportion of surveyed HCW who reported that they had received IPC training or education increased from 26.8%, in pre-intervention to 45.5% in the post-intervention study, *p* < 0.001 (Table 1). Nearly half (45.9%) of the post-intervention respondents reported receiving information on AMR during the 12 month period of project implementation. In both studies, almost all respondents (> 97%) acknowledged the need for more education and information on critical AMR. (Table 2a)

**Table 2a:** Knowledge of nurses, doctors, and laboratory personnel on infection control and AMR

Description	Pre- intervention *N* = 393	Post -intervention *N* = 420	Total *N* = 813	*P* value^‡^
	n (%)	n (%)	n (%)	
**1. Critical AMR organisms are reported from in the hospital**	(***n*** = **391**)	(***n*** = **418**)	(***n*** = **809**)	< 0.001
Agree/ Strongly agree	313 (80.1)	391 (93.5)	704 (87)	
Don’t know	68 (17.4)	22 (5.3)	90 (11.1)	
Disagree/ Strongly disagree	10 (2.6)	5 (1.2)	15 (1.9)	
**2. Need more education and information on critical AMR**	(***n*** = **393**)	(***n*** = **420**)	(***n*** = **813**)	0.94
Agree/ Strongly agree	391 (99.5)	418 (99.5)	809 (99.5)	
Don’t know	0	0	0	
Disagree/ Strongly disagree	2 (0.5)	2 (0.5)	4 (0.5)	
**3. Critical AMR infections can be transmitted from one patient to another**	(***n*** = **336**)	(***n*** = **366**)	(***n*** = **702**)	< 0.001
Agree/ Strongly agree	306 (91.1)	355 (97.0)	661 (94.2)	
Don’t know	24 (7.1)	3 (0.8)	27 (3.8)	
Disagree/ Strongly disagree	6 (1.8)	8 (2.2)	14 (2.0)	
**4. Critical AMR infections can be transmitted through shared equipment**	(***n*** = **340**)	(***n*** = **368**)	(***n*** = **708**)	< 0.001
Agree/ Strongly agree	292 (85.9)	352 (95.7)	644 (91.0)	
Don’t know	35 (810.3)	8 (2.2)	43 (6.1)	
Disagree/ Strongly disagree	13 (3.8)	8 (2.2)	21 (3.0)	
**5.Critical AMR infections can be transmitted to patients by staff**	(***n*** = **342**)	(***n*** = **368**)	(***n*** = **710**)	0.01
Agree/ Strongly agree	307 (89.8)	351 (95.4)	658 (92.7)	
Don’t know	21 (6.1)	8 (2.2)	29 (4.1)	
Disagree/ Strongly disagree	14 (4.1)	9 (2.4)	23 (3.2)	
**6.Understand what standard precautions are and know how to use them to prevent the spread of critical AMR infections**	(***n*** = **387**)	(***n*** = **417**)	(***n*** = **804**)	< 0.001
Agree/ Strongly agree	333 (86.1)	392 (94.0)	725 (90.2)	
Don’t know	40 (10.3)	16 (3.8)	56 (7.0)	
Disagree/ Strongly disagree	14 (3.6)	9 (2.2)	23 (2.9)	
**7.Understand what TBP are and know how to use them to prevent the spread of critical AMR infections**	(***n*** = **341**)	(***n*** = **368**)	(***n*** = **709**)	0.001
Agree/ Strongly agree	299 (87.7)	350 (95.1)	649 (91.5)	
Don’t know	31 (9.1)	11 (3.0)	42 (5.9)	
Disagree/ Strongly disagree	11 (3.2)	7 (1.9)	18 (2.5)	
**8.Hand washing or use of ABHR can reduce the spread of critical AMR infections**	(***n*** = **393**)	(***n*** = **420**)	(***n*** = **813**)	0.10
Agree/ Strongly agree	381 (96.9)	415 (98.8)	796 (97.9)	
Don’t know	6 (1.5)	4 (1.0)	10 (1.2)	
Disagree/ Strongly disagree	6 (1.5)	1 (0.2)	7 (0.9)	
**9. I feel confident of my knowledge of infection prevention and control**	(***n*** = **337**)	(***n*** = **365**)	(***n*** = **702**)	0.05
High (score of 4–5)	193 (57.3)	235 (64.4)	428 (61.0)	
Low (score 1–3)	144 (42.7)	130 (35.6)	274 (39.0)	
**10. I know what to do if a patient with CAR infection is admitted in the ward.**	(***n*** = **334**)	(***n*** = **362**)	(***n*** = **696**)	0.002
High (score of 4–5)	190 (56.9)	248 (68.5)	438 (62.9)	
Low (score of 1–3)	144 (43.1)	114 (31.5)	258 (37.1)	

‡Comparison of the pre- and post-intervention findings, ABHR = Alcohol-based hand rub, TBP = transmission-based precautions

### Knowledge

Most HCW across all categories were aware that critical AMR organisms had been identified in CWMH (80.1% pre- and 93.5%, post-intervention *p* < 0.001, respectively) (Table 2a). The majority of nurses and doctors (> 85% in pre- and ≥ 95% in post-intervention) knew that AMR organisms can be transmitted from one patient to another, via HCW contaminated hands to patients, as well as via contaminated equipment. Similarly, most HCW knew that standard precautions (86.1% in pre- and 94.0% in post-intervention, *p* < 0.001) and hand hygiene (96.6% in pre- and > 98.8% in post-intervention, *p* = 0.10) help to prevent the spread of AMR infections. Among nurses and doctors, 87.7% in pre- and 95.1% in post-intervention (p = < 0.001) knew about TBP for AMR prevention. Self-rated knowledge significantly increased post-intervention, where 68.5% of nurses and doctors reported to know what IPC practices were needed for patients with critical AMR infections compared to 56.9% at pre-intervention (*p* = 0.002). Staff confidence in their IPC knowledge improved over time (57.3% in pre- and 64.4% in post-intervention, *p* = 0.05).

Among laboratory personnel, knowledge about critical AMR and carbapenemase producing Enterobacterales detection was higher in post-intervention (65.3% and 79.2%, *p* = 0.10 and 57.1% and 67.3% *p* = 0.03, respectively, Table 2b). In both studies, fewer than half of the staff were aware of the procedures to follow after the identification of critical AMR organisms. Knowledge on the use of genomics for understanding transmission pathways of AMR organisms and the procedures to store bacterial isolates for whole genome sequencing remained unchanged (Table 2b).

**Table 2b:** Knowledge of laboratory personnel on infection control and AMR

Description	Pre-intervention *N* = 49	Post-intervention *N* = 49	Total *N* = 98	*P* value^‡^
	*n* (%)	*n* (%)	*n* (%)	
**1. Staff have adequate knowledge to detect critical AMR organisms**	(***n*** = **49**)	(***n*** = **48**)	(***n*** = **97**)	0.10*
Agree/ Strongly agree	32 (65.3)	39 (79.2)	70 (72.2)	
Don’t know	8 (16.3)	8 (16.7)	16 (16.5)	
Disagree/ Strongly disagree	9 (18.3)	2 (4.1)	11 (11.3)	
**2. Know how to detect carbapenemase producing** ***Enterobacterales***	(***n*** = **49**)	(***n*** = **49**)	(***n*** = **98**)	*0.03*
Agree/ Strongly agree	28 (57.1)	33 (67.3)	61 (61.0)	
Don’t know	1 (2.0)	6 (12.2)	7 (7.1)	
Disagree/ Strongly disagree	20 (40.8)	10 (20.4)	30 (30.6)	
**3.Know procedures to follow in case of identification of critical AMR organisms**	(***n*** = **48**)	(***n*** = **48**)	(***n*** = **96**)	*0.22*
Agree/ Strongly agree	17 (35.4)	24 (50.0)	41 (42.7)	
Don’t know	18 (37.5)	17 (34.4)	35 (36.5)	
Disagree/ Strongly disagree	13 (27.1)	7 (14.6)	20 (20.8)	
**4. Genomics helps to understand transmission patterns of critical AMR infections**	(***n*** = **48**)	(***n*** = **47**)	(***n*** = **95**)	*0.22*
Agree/ Strongly agree	31 (64.6)	32 (68.1)	63 (66.3)	
Don’t know	14 (29.2)	15 (31.9)	29 (30.5)	
Disagree/ Strongly disagree	3 (6.3)	0	3 (3.2)	
**5. Know procedure to store isolates for genomics testing**	(***n*** = **49**)	(***n*** = **48**)	(***n*** = **97**)	*0.91*
Agree/ Strongly agree	14 (28.6)	12 (25.0)	26 (26.8)	
Don’t know	24 (49.0)	24 (50.0)	48 (49.5)	
Disagree/ Strongly disagree	11 (22.4)	12 (25.0)	23 (23.7)	
**6. Information on critical AMR infection is readily to laboratory staff**	(***n*** = **49**)	(***n*** = **49**)	(***n*** = **98**)	*0.45*
Agree/ Strongly agree	20 (40.8)	26 (53.1)	46 (46.9)	
Don’t know	14 (28.6)	10 (20.4)	24 (24.5)	
Disagree/ Strongly disagree	15 (30.6)	13 (30.6)	28 (28.6)	

‡Comparison of the pre and post intervention findings, *Fisher exact test

### Attitudes

In both studies, almost all nurses and doctors agreed that it is important to collect data on critical AMR in CWMH (98.5% in pre- and 98.9% in post-intervention) and reported their willingness to change their work environment to prevent or control critical AMR infections (84.2% in pre- and 84.8% in post-intervention, *p* = 0.13) (Table 3). Over 90% of HCW who responded in both time periods reported that they believed overcrowding in CWMH increased the spread of AMR infections. Most HCW reported that they thought critical AMR organisms were common among hospitalised patients (74.1 in pre- and 81.1% in post-intervention, *p* = 0.06) and recognised that AMR infections could also occur in community settings (78.4% in pre- and 86.4% in post-intervention, *p* = 0.002) however less than a quarter of HCW (20.1% in pre and 22.2% in post-intervention) were aware that critical AMR can be a problem among international travelers. Most laboratory staff felt comfortable working in the laboratory where critical AMR organisms are processed (79.6 in pre- and 75.5% in post-intervention, *p* = 0.27). Among nurses and doctors the proportions were lower (61.9% in pre- and 63.5% in post-intervention, *p* = 0.74) compared to laboratory staff. In both time periods, around 30% reported that they were not comfortable working in wards or ICUs where patients with infections due to critical AMR organisms are admitted. In total, 94% of nurses and doctors recognised low risk of transmission of critical AMR to staff when caring for patients with AMR organisms. Significantly more nurses and doctors believed that they had adequate knowledge for critical AMR IPC in the post-intervention time period (63.4% in pre- and 81.4% in post-intervention, < 0.001).

**Table 3:** Attitudes of nurses, doctors, and laboratory personnel responses on infection control and AMR

Description	Pre-intervention *N* = 393	Post-intervention *N* = 420	Total *N* = 813	*P* value^‡^
	n (%)	n (%)	n (%)	
**1. Concerned about risks to staff when caring for patient with critical AMR infection**	(***n*** = **342**)	(***n*** = **371**)	(***n*** = **713**)	*0.64*
Agree/ Strongly agree	13 (3.8)	14 (3.8)	27 (3.8)	
Don’t know	9 (2.6)	6 (1.6)	15 (2.1)	
Disagree/ Strongly disagree	320 (93.6)	351 (94.6)	671 (94.1)	
**2.Feel comfortable working in wards/ICU where patients with critical AMR are admitted**	(***n*** = **341**)	(***n*** = **370**)	(***n*** = **711**)	*0.74*
Comfortable /Very comfortable	211 (61.9)	235 (63.5)	446 (62.7)	
Don’t know	26 (7.6)	23 (6.2)	49 (6.9)	
Not comfortable/ Very uncomfortable	104 (30.5)	112 (30.3)	216 (30.4)	
**3.Feel comfortable working in laboratory where critical AMR organisms are processed**	(***n*** = **49**)	(***n*** = **49**)	(***n*** = **98**)	0.27*
Comfortable /Very comfortable	39 (79.6)	37 (75.5)	76 (77.6)	
Don’t know	6 (12.2)	3 (6.1)	9 (9.2)	
Not comfortable/ Very uncomfortable	4 (8.2)	9 (18.4)	13 (13.3)	
**4. Willing to change work environment to prevent or control critical AMR infections**	(***n*** = **342**)	(***n*** = **368**)	(***n*** = **710**)	*0.13*
Agree/ Strongly agree	288 (84.2)	312(84.8)	600 (84.5)	
Don’t know	15 (4.4)	26 (7.1)	41 (5.8)	
Disagree/ Strongly disagree	39 (11.4)	30 (8.2)	69 (9.7)	
**5. Think critical AMR organisms can occur in community settings in Suva/surrounding areas**	(***n*** = **389**)	(***n*** = **418**)	(***n*** = **807**)	*0.002*
Agree/ Strongly agree	305 (78.4)	361 (86.4)	666 (82.5)	
Don’t know	50 (12.9)	43 (10.3)	93 (11.5)	
Disagree/ Strongly disagree	34 (8.7)	14 (3.3)	48 (5.9)	
**6. Think critical AMR organisms are mostly a problem in hospitalised people**	(***n*** = **390**)	(***n*** = **418**)	(***n*** = **808**)	*0.06*
Agree/ Strongly agree	289 (74.1)	339 (81.1)	628 (77.7)	
Don’t know	32 (8.2)	25 (6.0)	57 (7.1)	
Disagree/ Strongly disagree	69 (17.7)	54(12.9)	123 (15.2)	
**7. Think critical AMR organisms are mostly a problem with international travellers**,** rarely found in local people**	(***n*** = **388**)	(***n*** = **419**)	(***n*** = **807**)	0.54
Agree/ Strongly agree	78 (20.1)	93 (22.2)	171 (21.2)	
Don’t know	97 (25.0)	92 (22.0)	189 (23.4)	
Disagree/ Strongly disagree	213 (54.9)	234 (55.8)	447 (55.4)	
**8. Believe overcrowding in the hospital ICU and wards increases the spread of critical AMR infection**	(***n*** = **343**)	(***n*** = **370**)	(***n*** = **713**)	0.003*
Agree/ Strongly agree	319 (93.0)	363 (98.1)	682 (95.7)	
Don’t know	17 (5.0)	4 (1.1)	21 (2.9)	
Disagree/ Strongly disagree	7 (2.0)	3 (0.8)	10 (1.4)	
**9. Think to have adequate knowledge about critical AMR infection prevention and control**	(***n*** = **339**)	(***n*** = **370**)	(***n*** = **709**)	< 0.001
Agree/ Strongly agree	215 (63.4)	301 (81.4)	516 (72.8)	
Don’t know	25 (7.4)	15 (4.1)	40 (5.6)	
Disagree/ Strongly disagree	99 (20.2)	54 (14.6)	153 (21.6)	
**10.Think it is important to collect data on critical AMR in this hospital**	(***n*** = **343**)	(***n*** = **367**)	(***n*** = **710**)	-
Agree/ Strongly agree	338 (98.5)	363 (98.9)	701 (98.7)	
Don’t know	2 (0.6)	4 (1.1)	6 (0.8)	
Disagree/ Strongly disagree	3 (0.9)	0	3 (0.4)	

‡Comparison of the pre and post intervention findings, *Fisher Exact test

### Readiness

With regard to readiness, 41.3% of respondents in the pre- and 52.3% in the post-intervention study (*p* = 0.01) agreed that the hospital laboratory had adequate capacity to detect critical AMR organisms (Table 4). In both studies, nearly half of the doctors and nurses reported that the hospital did not have adequate capacity to respond to an outbreak of critical AMR infections (44.0% in the pre- and 51.9% in the post-intervention, ( *p* = 0.01) and reported that there were insufficient resources for management of patients with critical AMR infections (38.9% in pre- and 44.4% in post-intervention, *p* = 0.01). The proportion of HCW who were aware of AMR surveillance and reporting systems increased from 51.7% in the pre-intervention to 66.3% in the post-intervention, *p* < 0.001. Similarly, in the post-intervention study, more nurses and doctors reported that data on critical AMR organisms was readily available to them (29.4% in pre- and 52.4% in post-intervention cohorts, *p* < 0.001).

**Table 4:** Readiness of nurses, doctors, and laboratory personnel on AMR

Description	Pre-intervention *N* = 393	Post-intervention *N* = 420	Total *N* = 813	*P* value^‡^
	*n* (%)	*n* (%)	*n* (%)	
**1. Hospital has adequate laboratory capacity to detect critical AMR organisms**	(***n*** = **390**)	(***n*** = **419**)	(***n*** = **809**)	*0.01*
Agree/ Strongly agree	161 (41.3)	219 (52.3)	380 (47.0)	
Don’t know	122 (31.3)	114 (27.2)	236 (29.2)	
Disagree/ Strongly disagree	107 (27.4)	86 (20.5)	193 (23.9)	
**2. Hospital has adequate capacity to respond to outbreak of critical AMR infections**	(***n*** = **343**	(***n*** = **368**)	(***n*** = **711**)	*0.01*
Very adequate/ adequate	114 (33.2)	125 (33.4)	239 (33.6)	
Don’t know	78 (22.7)	52 (14.1)	130 (18.3)	
Not adequate /no capacity	151 (44)	191 (51.9)	342 (48.1)	
**3. Surveillance and reporting systems are in place to understand the burden of critical AMR infection**	(***n*** = **385**)	(***n*** = **415**)	(***n*** = **800**)	*< 0.001*
Agree/ Strongly agree	199 (51.7)	275 (66.3)	474 (59.3)	
Don’t know	135 (35.1)	98 (23.6)	233 (29.1)	
Disagree/ Strongly disagree	51 (13.2)	42 (10.1)	93 (11.6)	
**4. Adequate critical AMR surveillance data is readily available for doctors and nurses**	(***n*** = **339**)	(***n*** = **360)**	(***n*** = **699**)	*< 0.001*
Agree/ Strongly agree	100 (29.5)	195 (52.4)	295 (42.2)	
Don’t know	72 (21.2)	59 (16.4)	131 (18.7)	
Disagree/ Strongly disagree	167 (49.3)	106 (29.4)	273 (39.1)	
**5. Hospital has adequate resources to treat patients with critical AMR**	(***n*** = **342**)	(***n*** = **367**)	(***n*** = **709**)	*0.01*
Very good/good	149 (43.6)	168 (45.8)	317 (44.7)	
Don’t know	60 (17.5)	36 (9.8)	96 (13.5)	
Poor/very poor	133 (38.9)	163 (44.4)	296 (41.7)	

‡Comparison of the pre and post intervention findings

## Discussion

This study assessed the knowledge, attitudes, and readiness about critical AMR and IPC among 813 different professional staff including doctors, nurses, and laboratory personnel in Fiji’s main tertiary hospital. It found improvements in knowledge and attitudes to manage this problem after a dedicated educational campaign, but importantly more work is needed to reach high levels of organisational readiness for outbreaks in particular. Interestingly this study happened over a time a significant movement of nursing workforce in Fiji [27, 28]. As a result, a more junior nursing staff cohort participated in the post-intervention study. However, the education and training program was still associated with staff reporting feeling better prepared to manage critical AMR.

HCW awareness of IPC is of paramount importance for effectively addressing the burden of AMR. In our study, HCW reported good knowledge of standard and TBP as well as hand hygiene. These findings contrast with studies in India [12] and Ethiopia [13] where only 27% and 41% of HCW reported understanding TBP and hand hygiene, respectively. The results from Fiji were closer to a multicounty study in Europe [29] (86.9%) and slightly higher than a Zambian study (61%) among nurses [14].

This study demonstrated significant improvements in HCW knowledge following targeted interventions. Nearly half of post-intervention participants reported receiving IPC and AMR information and education. At post-intervention 93.5% of surveyed HCW were aware that critical AMR organisms had been reported in the hospital. More nurses and doctors reported that they were confident in their IPC knowledge and knew what to do for patients with critical AMR infections. This awareness is a fundamental step toward implementing effective containment measures. These improvements are likely to reflect the positive impact of the twelve months of integrated education and training provided by the intervention. There were some areas such as laboratory staff knowledge about surveillance, and genomics that likely need further dedicated attention.

Interestingly although HCW acknowledged AMR can occur in hospitals and in the community, many respondents believed AMR is not a problem among international travellers. Studies have shown travel to AMR prevalent countries increases the risk of colonisation with MDRO [30, 31] or acquiring an AMR infection if accessing medical facilities [32]. Fiji has an overseas medical referral system to countries where the AMR burden is high [33] and repatriates many patients to CWMH after these visits. More education is needed to raise awareness on the increased risk of AMR among returning travellers and patients. CWMH is developing standardised procedures to promptly identify, and screen patients deemed to be at high-risk for colonisation or infection because of overseas travel or treatment.

Adoption of globally recommended IPC measures including contact precautions, hand hygiene and appropriate environmental cleaning is effective in reducing the spread of critical AMR organisms in health care settings [10, 34]. The risk of transmission of CRO to HCW is believed to be minimal [10]. However, in both time periods, around one third of nurses and doctors expressed their discomfort at working with patients with critical AMR infections. Our study did not investigate the reasons for HCW’s concerns however, provision of adequate and uninterrupted supply of personal protective equipment, environmental cleaning and disinfection products, and hand hygiene consumables are important as well as ongoing education and proactive positive attitudes of staff.

Translation of knowledge and attitudes to changes in practice is a major challenge [26, 29, 35–37]. Our study didn’t investigate IPC practice. But contact precautions compliance audits conducted during the project implementation showed poor practices such as with hand hygiene compliance and use of PPE (Additional file 1 Table 3). Individual and group feedback were given to staff and IPC team throughout the project implementation so that local leaders (particularly nursing and ICU team leaders) were made aware of the performance of their ward in audits. Previous studies have demonstrated that education and training of HCW on IPC could improve IPC practices and reduce HAI [38] and the incidence of AMR [39, 40]. However, multiple confounding factors influence IPC compliance, including shortages of consumables, poor infrastructure, and high staff workloads [29, 35, 36, 41, 42]. In Fiji, a large number of HCW including nurses have migrated overseas or joined the private health sector since 2022 [27, 28]. With the departure of experienced HCW, the posts will often be filled by junior staff. Hence there is a need for ongoing education and capacity building activities to sustain improvement. Further major challenges for effective IPC in CWMH include inadequate equipment and infrastructure [19, 20]. Ongoing attention to address these barriers is needed to mitigate the risk and impact of critical AMR.

Most HCW respondents identified insufficient hospital capacity for laboratory detection, surveillance, outbreak response and treatment of critical AMR organisms as key ongoing gaps needing further attention. These findings are similar to previous studies in the Western Pacific region which highlighted a lack of national AMR surveillance programs (54.8%) and insufficient laboratory capacity for AMR detection (51.1%) [23]. Outbreak response requires coordinated systems, which local clinicians are working to build in Fiji. Access to last line antimicrobials to treat these infections is challenging, and many of these drugs are costly which may pose major difficulties for low- and middle-income countries.

The study findings should be interpreted in view of its limitations. Firstly, we used a convenience sampling method whereby readily available HCW were invited to participate. Also due to the departure of many senior nurses in 2022 and 2023, the post-intervention study enrolled more junior nurses compared to pre-intervention study. These two factors may have introduced selection bias which may have affected our results. Secondly, our results are based on self-reported responses, and we are unable to rule out possible over or under reporting. Thirdly, our studies did not investigate IPC practices, therefore improvement in knowledge and attitudes in the post intervention study does not necessarily signify better IPC practices as a result of the project’s capacity building activities. Lastly, the studies were conducted in one of the tertiary hospitals with high rate of AMR therefore results cannot be generalised for the whole of Fijian facilities.

In conclusion, our data revealed good AMR and IPC knowledge and attitudes. However, staff perceived that surveillance, readiness for outbreak response and treatment of patients with critical AMR organisms were inadequate. Improving laboratory and IPC capabilities for critical AMR in CWMH will require sustained and multisectoral interventions with strong administrative commitment. Such commitment should include recognition of AMR and IPC as priorities and availing the required human, financial and material resources needed to promote good IPC practices. Strengthening AMR governance requires establishing guidelines and standardisation for AMR surveillance, reporting and outbreak response. Integration of AMR and IPC topics into the continuing medical and nursing education will help to enable sustained HCW training and education.

## Electronic supplementary material

Below is the link to the electronic supplementary material.

Supplementary Material 1

## Data Availability

The datasets supporting the conclusions of this article are included within the article and its additional file (Additional file 1).

## Abbreviations

ABHR: Alcohol-based hand rub
AMS: Antimicrobial stewardship
AMR: Antimicrobial Resistance
CCU: Cardiac Care Unit
CRO: Carbapenem Resistant Organisms
CWMH: Colonial War Memorial Hospital
HAI: Healthcare Associated Infection
HCW: Healthcare Workers
ICU: Intensive Care Unit
IPC: Infection Prevention and Control
MDRO: Multi-drug Resistant Organisms
MDUPHL: Microbiological Diagnostic Unit Public Health Laboratory
Obs/Gyn: Obstetrics and Gynaecology
PPE: Personal Protective Equipment
SOP: Standard operating procedure
TBP: Transmission-based Precautions
WHO: World Health Organization
