# Supplementary material for: Knowledge, attitudes, and readiness about critical antimicrobial resistant organisms among healthcare workers at colonial war memorial hospital in Fiji: a pre and post intervention study
Source: Antimicrob Resist Infect Control. 2024 Sep 4;13:98. doi: 10.1186/s13756-024-01439-9 (PMC11373415; doi:10.1186/s13756-024-01439-9)
Supplement: Supplementary file 1 — Supplementary Material 1 [file 13756_2024_1439_MOESM1_ESM.docx]

**Additional file 1 Table 1: Training and education activities conducted in the Colonial War Memorial Hospital (1 August 2022 to 31 August 2023)**

**Structured training on infection prevention and control and critical antimicrobial resistance**

| **#** | **Date** | **Training topic** | **Staff cadre trained** | **Number trained** |
| --- | --- | --- | --- | --- |
| 1 | 17 October 2022 | Introduction to carbapenem-resistant organisms (CRO) - Standard operating procedure (SOP) | Nurse unit managers, IPC officers, doctors | 29 |
| 2 | 16 & 17 November 2022 | CRO - SOP and IPC  (with demonstrations of rectal swabbing, contact precautions at bed side and hand hygiene) | Ward nurses, doctors | 77 |
| 3 | 6 December 2022 | CRO case management for clinicians (ZOOM session) | Doctors | 15 |
| 4 | 21 March 2023 | CRO case management for clinicians | Doctors | 20 |
| 5 | 24 March 2023 | CRO case management | Doctors | 40 |
| 6 | 31 March 2023 | Contact precautions (CP) compliance audit  CP at bedside - demonstration | Nurses, Interns | 43 |
| 7 | 30 &31 August 2023 | Refresher training CRO management and IPC | IPC officer, clinical governance team | 5 |
| **Total number of HCW trained** | | | | **229** |

CRO = carbapenem- resistant organisms, SOP = Standard operating procedure, IPC = Infection prevention and control, CP= contact precautions, HCW = healthcare worker

**Training on laboratory procedures for antimicrobial resistant organism detection**

| **#** | **Date** | **Training topic** | **Staff cadre trained** | **Number trained** |
| --- | --- | --- | --- | --- |
| 1 | December 2022 | On the job training – Xpert Carba-R (qualitative PCR) and modified CIM | Laboratory technicians | 8 |
| 2 | January 2023 | On the job training – Xpert Carba-R (qualitative PCR) | Laboratory technicians | 5 |
| 3 | February & March 2023 | CHROMAgar media preparation and inoculation | Laboratory technicians | 7 |
| 4 | 30 August 2023 | Genomics training | Laboratory technicians  Doctors  IPC officers | 21 |
| **Total number of HCW trained** | | | | **41** |

**Ward level education sessions on infection prevention and control and critical antimicrobial resistance**

| **#** | **Date** | **Wards** | **Staff cadre trained** | **Number trained** |
| --- | --- | --- | --- | --- |
| 1 | 19 August 22 | Medical ward | Nurses | 6 |
| 2 | 23 August 22 | Medical ward | Nurses | 6 |
| 3 | 23 August 22 | Maternity ICU & ward | Nurses | 6 |
| 4 | 26 August 22 | Medical ward | Nurses | 6 |
| 5 | 31 August 22 | Pediatric Ward | Nurses | 10 |
| 6 | 9 September 22 | Surgical ward (operation theatre, OT post anesthesia, endoscopy, unit) | Nurses | 16 |
| 7 | 2 December 22 | Pediatric Ward | Nurses | 17 |
| 8 | 19 January 23 | Maternity ward | Nurses | 7 |
| 9 | 31 January 23 | Burns unit | Nurses | 6 |
| 10 | 24 February 23 | Surgical ward | Doctors | 42 |
| 11 | 7 February 23 | Pediatric ICU | Nurses | 19 |
| 12 | 8 February 23 | Various wards - ICU, IPC, surgical ward, post anesthesia, IPC, and hygiene staff | Nurses, hygiene staff | 18 |
| 13 | 24 February 23 | Neonatal ICU | Nurses | 17 |
| 14 | 2 March 23 | Anesthesia (hyperbaric team) | Nurses, doctors | 8 |
| 15 | 3 March 23 | Emergency department  Pediatric outpatient | Nurses  Intern doctors | 18 |
| 16 | 6 March 23 | CCU | Nurses | 9 |
| 17 | 10 March 23 | Surgical ward | Nurses | 13 |
| 18 | 4 April 23 | Various wards - ICU, CCU, IPC medical, maternity, OT, emergency, hygiene | Nurses, hygiene staff, ward assistants | 19 |
| 19 | 25 April 23 | Dental department | Dental team | 24 |
| 20 | 28 April 23 | Maternity | Nurses | 18 |
| 21 | 28 April 23 | OT | Ward assistant, hygiene staff | 11 |
| 22 | 3 May 23 | Medical ward | Nurses | 8 |
| 23 | 12 May 23 | Medical ward | Nurses, ward assistant, hygiene staff | 26 |
| 24 | 16 May 23 | Maternity ward | Nurses | 12 |
| 25 | 26 May 23 | ICU | Nurses | 14 |
| 26 | 1 June 23 | Maternity ICU | Nurses | 4 |
| 27 | 2 June 23 | Medical and surgical wards | Nurses | 12 |
| 28 | 8 June 23 | Neonatal ICU | Nurses, ward assistant | 13 |
| 29 | 20 June 23 | Hygiene team | Hygiene staff | 46 |
| 30 | 29 June 23 | ICU | Intern Nurses | 4 |
| 31 | 14 July 23 | ICU | Nurses, trainee nurses | 10 |
| 32 | 14 July 23 | Surgical ward | Nurses | 7 |
| 33 | 11 July 23 | CCU | Intern nurses | 4 |
| 34 | 21 July 23 | Clinical Governance team | Nurses | 6 |
| 35 | 1 August 23 | Medical ward | Doctors | 15 |
| 36 | 1 August 23 | Medical ward | Doctors | 15 |
| 37 | 2 August 23 | Maternity ward | Nurses Lecturers | 8 |
| 38 | 9 August 23 | Pediatric ward | Doctors | 34 |
| 39 | 10 August 23 | Dental department | Dental team | 22 |
| 40 | 17 August 23 | Medical ward | Medical interns | 13 |
| 41 | 17 August 23 | Pediatric ward | Nurses, ward assistants | 13 |
| 42 | 18 August 23 | OT | Nurses, ward assistants | 29 |
| 43 | 18 August 23 | Medical ward | Trainee intern nurses | 9 |
| **Total number of HCW trained** | | | | **620** |

OT= operation theatre, ICU= Intensive care unit, CCU=cardiac care unit, IPC infection prevention and control

**Additional file 1 Table 2: Contact precautions compliance audit in the Colonial War Memorial Hospital (March - October 2023)**

**Wards included in the contact precautions compliance audit**

| **Ward*** | **Number of observations** |
| --- | --- |
| Intensive care unit | 13 |
| Acute medical ward | 6 |
| Neonatal intensive care unit | 5 |
| Burns ward | 4 |
| Maternity ward | 4 |
| Paediatrics intensive care unit | 2 |
| Other wards | 2 |
| **Total** | **36** |

*Audits were conducted only in the wards where patients colonised or infected with carbapenem resistant organisms were admitted.

**Healthcare workers included in the contact precautions compliance audit**

| **Staff observed** | **Number** |
| --- | --- |
| Nurses* | 27 |
| Doctors | 6 |
| Physiotherapists | 2 |
| Hygiene staff | 1 |
| **Total** | **36** |

* Includes two student nurses and two intern nurses

**Contact precautions compliance audit**

| **Activities** | **% Compliant** | **Number compliant / total observation** |
| --- | --- | --- |
| **PPE donning** |  |  |
| HH before donning | 55 | 17/21 |
| Don gown correctly | 69 | 22/32 |
| Don gloves correctly | 67 | 22/33 |
| Correct PPE donning order | 64 | 21/33 |
| **PPE doffing** |  |  |
| Remove gloves | 71 | 25/35 |
| Perform HH | 31 | 11/31 |
| Remove gown | 60 | 21/35 |
| Perform HH | 59 | 20/34 |
| PPE disposed in clinical waster bin | 77 | 27/35 |
| Shared equipment cleaned after use | 42 | 8/19 |

PPE= Personal protective equipment, HH= Hand Hygiene

**Additional file 1 Table 3: Years of service of healthcare workers by professional category**

| **Description** | **Pre-intervention**  **(N=393)** | **Post -intervention**  **(N=420)** | **Total**  **N=813** | ***P* value‡** |
| --- | --- | --- | --- | --- |
|  | **n (%)** | **n (%)** | **n (%)** |  |
| **Nurses** | **(n=220)** | **(n=233)** | **(n=453)** | *0.004* |
| ≤12month | 7 (3.2) | 27 (11.6) | 34 (7.5) |  |
| 1-4 years | 68 (30.9) | 77 (33) | 145 (32) |  |
| 5-10 years | 61 (27.7) | 60 (25.8) | 121 (26.7) |  |
| >10 years | 84 (38.2) | 69 (29.6) | 153 (33.8) |  |
| **Doctors** | **(n=117)** | **(n=137)** | **(n=254)** | *0.55* |
| ≤12month | 22 (18.8) | 62 (14.6) | 42 (16.5) |  |
| 1-4 years | 24 (20.5) | 23 (16.8) | 47 (18.5) |  |
| 5-10 years | 39 (33.3) | 56 (40.9) | 95 (37.4) |  |
| >10 years | 32(27.4) | 38 (27.7) | 70 (27.6) |  |
| **Laboratory personnel** | **(n=49)** | **(n=48)** | **(n=97)** | *0.08* |
| ≤12month | 6(12.2) | 15(31.3) | 21 (21.6) |  |
| 1-4 years | 16 (32.7) | 12 (25) | 28 (28.9) |  |
| 5-10 years | 13 (26.5) | 14 (29.2) | 27 (27.8) |  |
| >10 years | 14 (28.6)) | 7 (14.6) | 21 (21.6) |  |

‡Comparison of the pre- and post-intervention findings
